# Ethical challenges around thirst in end-of-life care –experiences of palliative care physicians

**DOI:** 10.1186/s12910-023-00943-8

**Published:** 2023-08-09

**Authors:** Maria Friedrichsen, Caroline Lythell, Nana Waldréus, Tiny Jaarsma, Helene Ångström, Micha Milovanovic, Marit Karlsson, Anna Milberg, Hans Thulesius, Christel Hedman, Anne Söderlund Schaller, Pier Jaarsma

**Affiliations:** 1 Palliative Education and Research Centre, Vrinnevi hospital, Norrköping, Sweden; 2 Department of Health, Medicine and Caring Sciences, Linköping University, Linköping, Sweden; 3 Department of Neurobiology, Care Sciences and Society, Division of Nursing, Karolinska Institutet, Huddinge, Sweden; 4 Theme Inflammation and Aging, Karolinska University Hospital, Huddinge, Sweden; 5 Department of Internal Medicine, Vrinnevi hospital, Norrköping, Sweden; 6 Department of Medicine and Optometry, Faculty of Health and Life Sciences, Linnaeus University, Kalmar, Sweden and Region Kronoberg, Växjö, Sweden; 7 Department of Molecular Medicine and Surgery, Karolinska Institutet, Stockholm, Sweden; 8 R & D Department, Stockholms Sjukhem Foundation, Stockholm, Sweden; 9 Pain and Rehabilitation Centre, and Department of Health, Medicine and Caring Sciences, Linköping University, Linköping, Sweden; 10 Division of Society and Health, Department of Health, Medicine and Caring Sciences, Linköping University, Linköping, Sweden

**Keywords:** Palliative care, Thirst, Ethical challenges, Physicians, Thematic analysis

## Abstract

**Background:**

Thirst and dry mouth are common symptoms in terminally ill patients. In their day-to-day practice, palliative care physicians regularly encounter ethical dilemmas, especially regarding artificial hydration. Few studies have focused on thirst and the ethical dilemmas palliative care physicians encounter in relation to this, leading to a knowledge gap in this area.

**Aim:**

The aim of this study was to explore palliative care physicians’ experiences of ethical challenges in relation to thirst in terminally ill patients.

**Methods:**

A qualitative interview study with an inductive approach was conducted. Sixteen physicians working in four different specialised palliative care units and one geriatric care unit in different hospitals in Sweden were interviewed. The interviews were transcribed verbatim and analysed with a reflexive thematic analysis.

**Results:**

When presented with an ethical challenge relating to thirst, physicians attempted to balance benefits and harms while emphasizing respect for the patient’s autonomy. The ethical challenges in this study were: Starting, continuing or discontinuing drips; lack of evidence and traditions create doubt; and lack of interest and time may result in patient suffering.

**Conclusions:**

All physicians in this study reported that “Starting, continuing or discontinuing drips” was the main ethical challenge they encountered, where some were so accustomed to the decision that they had a standard answer ready to offer patients and families. Physicians reported that drips were a symbol of thirst quenching, life and survival but were not necessary in end-of-life care. Others questioned the traditions regarding thirst and emphasised drips in particular.

## Background

The goal of palliative end-of-life care for terminally ill patients is to prevent and relieve suffering as much as possible, but also to respect the patients’ wishes [1]. When patients near death, they may involuntarily or voluntarily stop eating and drinking (VSED) and have a prognosticated median survival time of seven days [2]. The most common symptoms before death were pain, fatigue, impaired cognitive functioning, and thirst and dry throat [2]. After patients VSED, health care professionals may not question patients decision but automatically interpret it as a natural dying process [3]. In palliative care (PC), death is seen as something natural [4, 5]; 63% of health care professionals in Switzerland perceived the VSED to be natural for a dying patient [6]. However, over the past decade, clinicians and ethicists have increasingly recognized VSED as a medically and ethically appropriate means to hasten death,[7] which stands in contrast to the WHO definition of PC, which intends neither to hasten death nor to postpone death [1]. In the final week of a patient’s life, there is a risk of thirst [8] as the patient may be unable to communicate his or her needs. This presents an ethical dilemma for health care professionals, especially when patients show signs of discomfort. Previous studies on thirst have been done 20–30 years ago, but these studies viewed thirst as a cause of suffering for terminally ill patients [9–11]. During the 25–48 h before death, patients drink on average 250 ml of fluids, but this varies significantly (25–1650 ml) [12].

PC physicians face a variety of ethical challenges. A common conceptual understanding of the term “ethical challenge” is lacking within health care research. In this study, we define ethical challenges as “when different values are in opposition to each other (within one or more persons). It occurs when a person does not know how to behave and act in the best way, feels doubt or discomfort or when he/she is uncertain with respect to how to interact in or react to the situation [13]. Ethical challenges will include, but are not limited to terms such as ethical issues, moral challenges, moral dilemmas, values, good/bad, right/wrong. Ethical challenges can be labelled as such either by authors or by participants [14]. We chose this definition to ensure that all health care professionals understand these related terms, which may in turn affect the ability of different readers to understand the intended meaning of the concept.

One of the most frequently identified ethical challenges is artificial nutrition and hydration [15]. A few studies have focused on artificial infusions to terminally ill patients. One study from a palliative context reported that SC infusions of max 1000 ml/24 h had a significant effect on nausea and thirst after 24 h, but not on thirst after 48 h [16]. However, a review reported that none of three studies, all published 20 or more years ago, demonstrated any impact of artificial hydration on the experience of thirst [17]. Other studies report a positive effect of reducing IV infusions, such as a relief in abdominal pain/distention, a decrease of peripheral oedema, nausea and dyspnoea as well as increased quality of life, global satisfaction, and a feeling of benefit [18, 19]. These studies followed the Japanese guidelines regarding hydration therapy, where the IV volume recommendations for the example of thirst varies from not receiving artificial hydration at all, to 500–1000 ml/day [20]. A recent study showed that IV infusion at 400 ml/24 hour did not prolong survival nor significantly improved the dehydration symptoms, but improved the quality of dying [21]. Other studies did not find any difference when administering IV infusions to improve delirium, quality of life, symptom burden, consciousness or survival [22–24]. A Cochrane review [25] concluded that there are few studies, with low quality, that examine the benefits and harms of the use of artificial hydration in PC.

In PC, the four bioethical principles described by Beauchamp and Childress are important [26]. The first principle is autonomy, “a norm of respecting and supporting autonomous decisions”. The second and the third principle are beneficence, “a group of norms pertaining to relieving, lessening, or preventing harm and providing benefits and balancing benefits against risks and costs the duty to act in patients’ best interests”, and nonmaleficence, “a norm of avoiding the causation of harm”. Finally, the fourth principle is justice, “a cluster of norms for fairly distributing benefits, risks, and costs”. In Sweden, a patient has the right to refuse treatment and to have his/her decisions respected as long as possible, but patients cannot request a treatment [27]. The goal of care and the decision to discontinue treatment are difficult issues that are often discussed in PC [28, 29].

The evidence regarding thirst in end-of-life care has been neglected in recent years. Therefore, it is important to increase the use of the knowledge PC physicians provide in relation to this phenomenon, as they encounter terminally ill patients on a daily basis. Due to a knowledge gap in relation to this phenomenon, a qualitative study is appropriate. This study is a part of a larger project, “The Thirst Project”, which studies thirst and related ethical challenges in end-of-life care from different perspectives, such as the perspectives of terminally ill patients, family members, health care personnel and the biomedical area. The aim of this study was to explore PC physicians’ experiences of ethical challenges in relation to thirst in terminally ill patients.

## Methods

### Design

A qualitative, reflexive thematic design with an inductive analysis according to Braun & Clarke [30, 31] was used. The study was guided by the Standard for reporting qualitative research (SRQR) [32].

### Sampling and setting

Data were collected in four different sized cities in Sweden, with populations between 44,000 and 1,000,000. All cities had advanced PC units. Purposeful sampling was used to achieve a participant mix of different geographic locations, genders and ages (Table 1). The inclusion criteria were as follows: working as a physician in specialised PC or geriatric care; at least five years’ experience working with end-of-life patients; fluent in Swedish. The head of the department or a senior physician in charge asked the physicians to participate.

**Table 1:** Demographic data of the participating physicians

*Gender*	
Male/female	13/3
**Age**	
M	52
Min-max	37–66
***Medical degree***	
Residents	
Specialists	3
Consultants	13
Specialised in*	
Palliative medicine	10
Geriatrics	6
Internal medicine	4
Oncology	4
General medicine	1
Gynaecology and obstetrics	1
Haematology	1

### Data collection

Data were collected in 2020–2022 using personal recorded interviews (n = 10) and telephone interviews (n = 6). The interviews lasted between 16 and 35 min, and a medical student (HÅ) with a special interest in PC and previous experience as a nurse in end-of-life care conducted ten physical interviews. The interviews were carried out in a secluded room at the hospital in each respective town. The medical student transcribed these interviews for educational purposes. Due to the COVID-19 pandemic, six interviews were carried out by telephone. Three interviews were conducted by a research nurse with an MSc, as well as experience within PC and interviewing, and three were conducted by a nurse with a PhD and extensive experience working with cancer patients and interviewing. A professional transcriber transcribed all six of these interviews verbatim. The interview guide addressed physicians’ experiences of caring for end-of-life patients and their views on thirst and ethical challenges (Table 2) (the data on thirst are reported elsewhere) when caring for the patient group. The question regarding ethical challenges was, “Do you think there are any ethical problems that may arise when patients at the end of life are thirsty”? Participants were asked to talk freely, and follow-up questions were occasionally asked in order to achieve greater clarity, for example, “Please explain; Please tell me more; and Why/Why not?; and How?”

**Table 2:** Interview guide

1. Have you thought about whether end-of-life patients can be thirsty? If so, how?
2. Do you think end-of-life patients suffer from thirst? Justify!
3. Do you have a policy in your unit regarding thirst? Why/why not?
4. What are your colleagues’ views on thirst in patients at the end of life?
5. Can there be any ethical problems with patients who are thirsty?
6. Do you check whether a patient at the end of life is thirsty? How do you assess whether a patient is thirsty? If yes, what checks do you make? If not, why not?
7. Do you do anything to quench the patient’s thirst? What do you do? What do you think works best/worst?
8. Have you ever discussed thirst with relatives of patients at the end of life? Tell me!
9. How do you think one could work to quench thirst?
10. Is there anything I have not asked about thirst that you think is important to share?

### Data analysis

A reflexive thematic analysis according to Braun & Clarke [30, 31] was used. The design identified, analysed and interpreted patterns of meaning from the qualitative data and used the results to report concepts and assumptions underpinning the data, which are presented in themes.

A six-step process guided the analysis [33], for example see Table 3.

**Table 3:** Examples of the analysing process

*Interview excerpts (interview number, side number and line number)*	*Generating initial codes of relevance to the research question*	Generating themes	Defining and naming themes	Questioning and reflection process from co-authors	Themes of meaning
I put on a drip to see what happens, just because the relatives argue that much, not because the patient needs it. 6: 7–8; 22–24, 1–3.	The pressure of the relatives makes the physician decide to provide a drip, which is not in line with the physician’s assessment of the patients’ needs.	The physician do something against his/her own assessment and starts a drip.	Starting a drip is an ethical challenge.	This physician risks hurting the patient just because he/she cannot withstand pressure from relatives or does not wish to hurt their feelings. This physician needs to prioritize his or her loyalties. The patient’s well-being should trump the relatives’ well-being.	Starting, continuing or discontinuing drips
We (physicians) assume that patients are not thirsty, without really knowing. 1:3;15.	The physician believe in working routines, but do not really have knowledge about thirst.	The physician question the lack of knowledge among him/her-self and other physicians.	The lack of evidence creates questioning about the working routines around thirst.	The perspective needs to be highlighted - is there a real lack of evidence, or do the informants perceive there is a lack of evidence, or do they acknowledge that they themselves are not well informed, even though there is existing evidence?	Lack of evidence and traditions create doubt.
If our palliative care unit is responsible for the care, then mouth care is a part of our protocol, of our care. On the other hand, if the patient is at home and the community service is involved, then they are responsible. It happens that they miss this. 8: 3;15–23.	In home care, there are different providers of oral care, who do not have the same routines regarding oral care as the specialised, which means they miss it.	There are providers of oral care that do not have the same routines for oral care, which means that they do not perform it.	Specialist palliative care vs. non-specialist palliative care	I think this is a strange ethical dilemma. It is not about the specialisation, but it is about leaving the care to others? Trusting your colleagues?	Lack of interest and time may result in patient suffering.

1. Familiarisation with the data, reading the whole dataset several times in order to become intimately familiar with the data.
2. Generating initial codes to produce clear labels containing pieces of information that may be of relevance to the research question.
3. Generating themes, where the coded data were reviewed and analysed to determine how different codes may be combined according to shared meanings to form distinctive themes or sub-themes.
4. Reviewing potential themes, where the quality, boundaries and meaningfulness of each potential theme were scrutinised.
5. Defining and naming themes and producing the report. Each theme and sub-theme were to be expressed in relation to both the dataset and the research question. Multiple extracts were used from the data items that inform a theme in order to convey the diversity of expressions of meaning across these data items.

Two authors (HÅ, MF) independently read all 16 transcripts and conducted coding and development of themes and sub-themes. The first author was a medical student and the second an associate professor with a PhD in palliative medicine with extensive experience of PC research and thematic analysis. Different coders were used in order to sense-check the ideas and explore alternative interpretations of the data. The final analysis was discussed in the research group until a consensus was reached. In order to further strengthen the credibility of the results, the findings were clarified using quotations from participants.

## Results

When physicians narrate about ethical challenges, one theme and three sub-themes emerged. The main theme was: Balancing benefits and harm and the three subthemes were: Starting, continuing or discontinuing drips (artificial hydration); Lack of evidence and traditions create doubt; and Lack of interest and time may result in patient suffering.

### Balancing benefits and harm

All physicians discuss ethical challenges around thirst from the standpoint of what the right thing to do is for the patient and what will cause harm. When making a decision, they emphasise the principle of patient autonomy, as well as maleficence and beneficence. Physicians attempted to solve the ethical challenges they encountered by balancing on two different poles and weighing these factors against each other (Table 4).

**Table 4:** Overview of the theme and subthemes regarding ethical challenges around thirst

**Balancing benefits and harm**
*Starting, continuing or discontinuing drips*
*Lack of evidence and traditions create doubt*
*Non-specialist palliative care may result in patient suffering*

#### Starting, continuing or discontinuing drips

Physicians reported that a common ethical challenge was that family members asked for or demanded drips for their relative, as they perceived that the patient was thirsty even though the physicians themselves held an opposing opinion. The physicians clarified that drips were a symbol of thirst quenching, life and survival but were not needed in end-of-life care. The physicians claimed that some family members were very focused on this issue and were very determined that drips were needed.

*The discussion with family members is often: “you are letting my mother or father starve or die of thirst” “(family member says) Why can’t you give drips if my mother or father cannot drink independently anymore?” I:3*.

Physicians felt that they could cope with this ethical challenge, but the main question for them was whether this was a benefit or harm to the patient. All physicians wanted to avoid harming the patient by providing fluids that they did not need, which could result in dyspnoea, oedema and terminal respiratory secretions. Then they collected information from the patient, the family and team members, so that it was possible to make an individual decision.

*Many times, they (the family) say that the patient is thirsty. Then we have to investigate dry mouth and thirst. If the patient is still urinating, then I can confidently tell them that there are fluids in the body and it is unlikely that the patient is thirsty. This is the content in every discussion in end of life. I:5*.

At the same time, the physicians acknowledged that these situations are demanding for the families, as they may have a hard time accepting that a patient is dying. Some reported that they had given drips, so called “compassion drips”, just to please the family members. In these cases, they tried to see it from the family’s point of view and sometimes agreed to provide a drip just to give the family time to understand and accept the situation. Then the physicians weighed the patient’s well-being against the family’s well-being. The physician-family meeting had to result in structured agreement, as they wanted everything planned regarding the compassion drip so that it would not harm the patient.

*To get the family member and the patient to understand. In addition, you have to understand their side as well, why they think the way they do and have their opinion. To use the discussion to reach a consensus on the patient’s care. I:6*.

*… In that case, I think it is more of a problem when family members perceive that the patients’ needs relate to thirst and are in need of a drip. Then it can be an ethical problem. I usually analyse whether this will hurt the patient or not. If I agree to put on a drip, then I have to evaluate in a day or two, to be sure that it will not hurt the patient, that it will not cause more rattles, for example. I:7*.

*This question (about drips) is always preconceived, and there are certain cases when you do not reach the family members, and then you have to put on a drip, just so that they do not feel that they have not done everything they can. I:14*.

However, prescribing a drip in the last phase of a patient’s life can also lead to a discussion or even a conflict with another team member who holds an opposing opinion.

*Sometimes you need to have a discussion with a nurse or colleague who does not at all think that this patient should have a drip. I:5*.

In some cases, when the patient arrived at the PC unit, they already had an ongoing drip that was prescribed from another clinic. Then the physicians had to determine how to approach the discussion around this, a discussion that demanded time and patience.

*Sometimes you get patients that already have a drip. Then I think it is impossible to say: “Hi, welcome to my ward, and we are going to discontinue the drip on day one”. That doesn’t work, you have to start a dialogue that takes time, to reach everyone, come closer together and talk to each other.// Of course, sometimes you give drips that are not useful, but it may even be good. I:11*.

Some physicians were so accustomed to the question of drips that they had a standard answer ready – that thirst was better relieved through proper mouth care. Others were more pro-active; they addressed questions about drips with the patient and family before they asked themselves.

*I usually precede all these discussions about thirst in end of life by talking to the patient and family before we are in the situation when the patient cannot take anything orally anymore. I:15*.

#### Lack of evidence and traditions create doubt

Several physicians described that another ethical challenge was that they did not have enough knowledge or scientific evidence regarding thirst. When patients are not able to express themselves any longer, they cannot be sure if the patient is thirsty, as they do not have any objective measurements to use for thirst. This made them feel doubtful. Some were concerned that they had not paid enough attention to thirst, but held on to old traditions that all patients suffered from dry mouth and not thirst, and drips were not needed, something that is traditionally taught in their PC clinic.

*There is some kind of tradition that you should not give drips in end of life, but sometimes you need it. I:5*.

Some physicians felt insecure regarding the scientific basis and in turn, the information that they provided to patients and their families about thirst, that patients in end of life did not feel thirst. The whole team gave this information, as this was part of the tradition in PC. Then they felt distress about not being honest with the patient and the family member, that patients did not feel thirst, when they did not know this for sure.

*You give out information that you cannot back up with scientific evidence. That is an ethical dilemma. You give information to family members who are worried and have this question. You calm them down with a half-truth, as we actually do not know. I:1*.

*An ethical question related to patients and family members who request drips; we think that we know that it is not useful or even causes harm (drips). However, the truth is that we do not know. I:2*.

*You try get in contact with the relatives; if rattles appear, then we have to discontinue (the drip). This can sometimes be very hard, as I feel that I do not have 100% knowledge regarding thirst in end of life. In addition, I describe that the thirst is in the mouth, if that is even true, but that is what I say. I:15*.

Other physicians thought about the unspecific symptoms in end of life, such as anxiety, restlessness, or confusion that could be a sign of thirst, but instead treated these symptoms by giving the patient sedative medication. Then it was impossible to know if the patient was thirsty or suffered from anything else, which the physicians reported to be frustrating, as they did not know what they were treating.

*If the patient is anxious because of thirst, and I reduce the worry with benzodiazepines and make the patient more paralysed by that action, I reduce the symptom worry, but not what really caused the worry, which might be thirst. That is an ethical dilemma. I:1*.

Some physicians claimed that other team members can have an extreme view regarding drips and thirst, and therefore always refused to give drips. That was an ethical challenge, as the discussion was more about holding on to traditions than adjusting the treatment to meet the needs of the individual patient. Instead, they reported that the more experience you have the more open and humble you are to the patient’s individual needs.

*If you are new in this area, so to say, it’s easy to become a little bit more fundamentalist and always say that you should not (give drips). The longer you have been in the field and the more experience you get, the more you become nuanced in these discussions, like on one side or the other… like that. Today, I think it is much easier to give drips to the patient than when I was new, when you are trying to learn how it all works. I:13*.

Some physicians had considered giving drips, but held back due to strong traditions, even though they thought a drip would reduce patient suffering.

*There have been discussions regarding the lack of evidence that treatment with drips does not help. My experience is that many are dry in their mouth, and those who can express themselves in their last days in life say that they are thirsty. Then I think about the guidelines that we use, that you should not give drips, but mouth care. This probably works very well for most patients, but sometimes I get a feeling that maybe they would need something more, they may be dehydrated. That they even would need some fluids, intravenous fluids. However, I do not know that for certain. I:15*.

#### Lack of interest and time may result in patient suffering

Some of the physicians expressed that another ethical challenge arose when they knew that the patient was dependent on professionals that had lack of interest or time to relieve thirst. In specialised PC, they relied on the nursing staff to provide mouth care according to the local guidelines at the end of life, and thereby quenching the patients thirst. In other contexts, some physicians had experienced that nursing staff did not have the time to do this or were less interested in providing this care. Then they reported feeling distressed, as patients may suffer harm.

*If our palliative care unit is responsible for the care, then mouth care is a part of our protocol, of our care. On the other hand, if the patient is at home and the community service is involved, then they are responsible. It happens that they miss this. I:8.*

*If you have health care staff around you all the time who can help moisten your mouth, then it probably works well. Nevertheless, if you live alone, which many persons do today, and are referred to the community service, who might be at your home seven times a day, but unfortunately you were not thirsty when they were there, no. Then there can be a lot of suffering. I:5.*

Physicians tried to address this ethical challenge by educating the health care staff in the non-specialist context, but they are still aware that these staff members may not have time to give patients something to drink or to moisten their mouth.

*At home, there are family members or community staff who are the ones who will provide the treatment, who will perform the mouth care. This can be of different quality. Then we guide them. The nurses and assistant nurses who visits the patient are those who provide this education. I:2.*

## Discussion

This study shows that there are three main ethical challenges around thirst in terminally ill patients: *Starting, continuing or discontinuing drips*, lack of evidence and traditions create doubt, and non-specialist palliative care may result in patient suffering.

Starting, continuing or discontinuing dripswas the main ethical challenge and was mentioned by all physicians, as they thought that infusions in end-of-life care would do harm to the patient, resulting in pulmonary oedema, dyspnoea or terminal respiratory secretions. This opinion has been supported in other studies that looked at withdrawing or withholding interventions [34]. In addition, one survey found that PC physicians did not believe that infusions would help symptoms such as thirst [35] but would rather increase upper respiratory tract secretions (85%), ascites (73%), physical discomfort (72%) and dyspnoea (62%) [36]. However, a review study [17] regarding infusions in end-of-life care found the opposite, where the majority of studies did not find higher rates of respiratory secretions, or evidence of dyspnoea. This comparison of physicians’ experiences in the current study and the evidence from the review study is a sensitive issue in many ways. It reveals a factual disagreement about the level of suffering that infusion will cause, as the scientific evidence is not in line with the experience of PC physicians. Based on the results of the present study, there is a strong tradition of withholding infusions in PC clinics in Sweden, and some PC physicians had doubts regarding this tradition and looked more positively on providing infusions than others. A recent study reported that PC physicians experienced a “fixed” attitude regarding assisted hydration being inappropriate for all dying people; the concept of a ‘good death’ was associated with the non-provision of assisted hydration [37]. Physicians described that it was important to be open-minded regarding infusions and always take individual choices. However, one could question whether physicians should provide a “compassion drip” on the family’s request. This is a question of choosing between loyalties, as it may be impossible to provide a “compassion drip” while keeping the promise “do no harm to the patient”. It becomes a question of being loyal to the family members versus the terminally ill patient. On the other hand, according to the WHO definition of PC [1], physicians should support family members as well. Compassion can be defined as a sensitivity to the suffering of self and others with a commitment to prevent it and relieve it [38]. Beauchamp and Childress [26] claim that compassion can also cloud judgment and impede rational responses.

A review study [17] found a limited amount of predominantly low-quality research evaluating the impact in the last days of life and a pressing need for well-designed studies that focus on patients specifically in the last days of life and incorporate outcome measures that consider patients’ concerns. In the current study, physicians mentioned the lack of evidence in this area and it is understandable that PC physicians feel frustration regarding this, as the available studies are difficult to compare. This is depending on different patient diagnoses, when patients participate in the study (last 24 h, last week or last month), what culture they are living in, and the prescribed amounts of fluids, study design and measurements used. This feeling of lack of evidence reported in the current study is supported by a study [39] about thirst in heart failure patients, where health care professionals described that they often felt powerless, realizing that their treatment methods were currently insufficient. Thirst was experienced as a very complex symptom, causing suffering in these patients.

In the current study, the ethical principles of beneficence/nonmaleficence presented challenges in relation to thirst, but the study also identified challenges related to deception and honesty, which may be a new finding in this area. Physicians in this study reported that they wanted to be sure that they were providing true information regarding thirst to patients and families, but were not sure that they did, as they were uncertain of the evidence and the traditions. In a study among surgeons, half-truths were used in the context of balancing hopefulness and honesty in their conversations when bad news was wrapped up in more positive findings [40]. According to the German philosopher Kant, [41] such behaviour would constitute a lie, since it would devastate confidence among humans. Alternatively, it is deception to prevent injury or to protect patient’s feelings. Another study on honesty among PC nurses showed that honesty was a virtue, a principle of good moral(42), which is more in line with Kant’s theory [41]. The PC physicians in the current study balance the different alternatives in facing these ethical challenges, as well as what they say to seriously ill patients. Previous studies [43, 44] have shown the balancing act between competing values; doing what is ethically right, to do what you believe to be in the best interests of the patient, to do what is required by law, and to avoid harm. One study showed that there are no perfect solutions for trajectories where the end point is death, there is a fine line between success and failure [44].

Honesty is something all humans should strive towards, but it may not be possible in all situations. Though there is a lack of sufficient evidence in an area, the PC physicians in the current study attempt to avoid saying, “We do not know”, as this may also raise questions of veracity. Beauchamp and Childress [26] discuss veracity as an obligation to loyalty, promise keeping and contract. In communication, there is an implied promise of truthfulness and avoidance of misleading each other. By entering into a relationship in PC, the patient can expect truthful information regarding prognosis and treatment, and the physician has the same right to truthful information from patients, since it is essential for trust in their relationship. Cautious management of medical information, which may include limited disclosure and even lying, is occasionally justified when veracity conflicts with other obligations, such as those of medical beneficence [26] .

The last theme “Lack of interest and time may result in patient suffering” is an important area where PC physicians see the problem in other organisational areas where a lack of knowledge may be the problem. One solution for this would be palliative care consultation teams that can be of assistance with bot education and bedside practise [45, 46]. However, nursing leadership, discussing nursing ethics and an active use of PC guidelines are important, so that the knowledge regarding patient needs at the end of life becomes clear.

This study showed that physicians take ethical challenges related to thirst very seriously in relation to communication and building relationships with patients and families. This has also been confirmed in another study, which showed that morality is partly about knowing how to build a relationship with patients and recognizing moral principles, while emphasising dialogue and communication [47] .

This study highlights the need for individual careful communication, where all risks and benefits are included. Patient and family members’ perspectives as well are their religious and cultural context are important to consider in this communication, as the value of a human life may be interpreted differently between professionals, patients, and family members. As there is a lack of evidence to guide the concerns about either giving or withholding artificial hydration, the professionals have to make a thorough assessment if patients are thirsty or not, for example assess symptoms of dehydration or anxiety. It is also important to ask both patients and families about their views around hydration, such as oral care as well as artificial hydration, as drinking as well as artificial hydration might be a symbol for life. The ethical principles of autonomy, beneficence, non-maleficence, and justice need to be applied when discussing these matters in the team.

### Strength and limitations

The choice of study design depended on the scarceness of qualitative research in this context and the purpose of the study.

There are several methodological limitations. Several interviewers conducted interviews, with a potential risk of methodological inconsistencies, which may influence the quality of the interviews. Data were analysed and discussed by a multidisciplinary team of researchers, which ensures scientific rigour. In this study, sixteen participants were included, and no new data emerged regarding the ethical challenges around thirst. However, a geographic spread was achieved, as we recruited physicians from four different PC units in Sweden. Although the study concerned PC and was conducted in Sweden, our findings may be transferable to other PC settings in countries with similar organisations.

To conclude, PC physicians experience ethical dilemmas regarding thirst when there is a request for infusions from terminally ill patients and/or families. When clinicians choose between different action alternatives to solve the ethical challenges, they need to collect information from different sources, such as biomedical data, experiences and opinions from patients, families and team members. Furthermore, it is important to share and explain knowledge to all involved parts so that everyone can participate. Reaching an agreement through dialogue and communication is also important, even though it is not always possible. Further studies in this field should focus on PC nurses and assistant nurses regarding the ethical challenges they experience in relation to thirst.

## Data Availability

The datasets generated and/or analysed during the current study are not publicly available due ethical restrictions but are available from the corresponding author on reasonable request.
